# Weak, Quiet Magnetic Fields Seen in the Venus Atmosphere

**DOI:** 10.1038/srep23537

**Published:** 2016-03-24

**Authors:** T. L. Zhang, W. Baumjohann, C. T. Russell, J. G. Luhmann, S. D. Xiao

**Affiliations:** 1 CAS Key Laboratory of Geospace Environment, University of Science and Technology of China, Hefei, China; 2 Space Research Institute, Austrian Academy of Sciences, Graz, Austria; 3 IGPP, University of California, Los Angeles, USA; 4 Space Sciences Laboratory, University of California, Berkeley, CA, USA

## Abstract

The existence of a strong internal magnetic field allows probing of the interior through both long term changes of and short period fluctuations in that magnetic field. Venus, while Earth’s twin in many ways, lacks such a strong intrinsic magnetic field, but perhaps short period fluctuations can still be used to probe the electrical conductivity of the interior. Toward the end of the Venus Express mission, an aerobraking campaign took the spacecraft below the ionosphere into the very weakly electrically conducting atmosphere. As the spacecraft descended from 150 to 140 km altitude, the magnetic field became weaker on average and less noisy. Below 140 km, the median field strength became steady but the short period fluctuations continued to weaken. The weakness of the fluctuations indicates they might not be useful for electromagnetic sounding of the atmosphere from a high altitude platform such as a plane or balloon, but possibly could be attempted on a lander.

The existence of a strong intrinsic magnetic field and the presence of time-varying fields can both be used to probe the electrical conductivity of a planetary crust^1^,^2^. Based on the Venus near-equatorial magnetic field surveyed down to 150 km altitude by the Pioneer Venus Orbiter (PVO), it is generally accepted that Venus has no significant global intrinsic field^3^,^4^. Although strong variable fields are present in the Venus ionosphere, these may be largely spatial variations, fossil fields impressed by slowly changing interplanetary magnetic fields “played back” at higher frequencies by the spacecraft’s rapid motion through the ionosphere. If so, then the fields at the surface of Venus resulting from the diffusion of the ionospheric magnetic field into the Venus atmosphere might be significantly weaker than in the ionosphere and much quieter^5^.

The Venus Express mission, designed to complement PVO, was inserted into an elliptical orbit with periapsis over north pole^6^,^7^. Initially, its periapsis was close to 78˚ latitude north and 250 km altitude. Later, the spacecraft periapsis moved to higher latitude over the pole and then to lower latitudes. Periapsis altitude was variable but typically remained above 160 km. A joint analysis of the Pioneer Venus and Venus Express measurements at low altitude within the ionosphere revealed that the magnetic field was horizontal on the dayside and expanded both into the wake at low altitudes and away from the wake at high altitudes^8^. During the last days of the Venus Express mission May – July 2014, an aerobraking campaign was performed. The changes of the spacecraft orbit allowed the periapsis to go as low as 129.7 km in altitude, which is well below the main peak ionosphere altitude of ~140 km^9^,^10^. Note that the lowest altitude measured by the PVO magnetometer was 150 km.

Figure 1 shows examples of the magnetic field strength observed near periapsis by the Venus Express magnetometer^11^ from 5 consecutive passes of those low altitude orbits. The time series of 1 sec resolution of magnetic field data are displayed for five minutes before and after the periapsis. The range of solar zenith angle (SZA) was 93.6° to 95.3°, and the range of periapsis altitude was 129.7 to 130.7 km for these passes. As illustrated in Fig. 1, a strikingly persistent feature, i.e., a weak and quiet field, is present in all passes for the 2 minutes around periapsis. While the Venusian ionosphere exhibits two magnetic states depending on the solar wind dynamic pressure conditions: either a magnetized ionosphere with large-scale horizontal magnetic fields; or an unmagnetized ionosphere with numerous small-scale thin structures, so-called flux ropes^12^,^13^. The magnetic field below 140 km is relatively weak and less fluctuating.

In order to illustrate this point more clearly, we display in Fig. 2 the altitude profiles of the magnetic field measurements on 33 passes, from June 6 to July 12, 2014. Here are shown altitude profiles of the magnetic field strength whenever the Venus Express periapsis was below 150 km altitude. All together, 66 altitude profiles (counting inbound and outbound separately for each pass) are displayed in Fig. 2a. We also calculated the standard deviation of the magnetic field time series for each 12 second interval and show them in panel b of Fig. 2. The green lines on panels a and b show the median field strength and fluctuation amplitude at each altitude. Panel c shows the ratio of the median short period fluctuation amplitude to the median field strength. The median magnetic field strength decreases with decreasing altitude from 150 to 140 km altitude, where lies the peak density of the ionosphere^9^,^10^. Below this altitude, the median field does not decrease. The short period fluctuation amplitude continues to drop to close to 130 km altitude, as emphasized in panel c, that shows the ratio of the fluctuation amplitude to the field strength.

The leveling off of the median field at the constant value near 7 nT at 140 km altitude is to be expected because this is the lowest level at which we expect strong electric currents to flow. At high altitudes, the spacecraft can cross current layers between regions of varying magnetic field directions and magnitudes but in the region below 140 km, the magnetic field is essentially derivable from solely a scalar potential since no current layers are present here. This observation is consistent with the earlier PVO-era models constructed to simulate the magnetic field down to an altitude of 120 km^13^,^14^.

The field strength below 140 km altitude has an average of 6.5 nT and median of 6.4 nT, and the field fluctuation δB has an average of 1.3 nT and median of 1.1 nT. This small and quiet polar atmospheric magnetic field, which we might further postulate is similar at the surface, has implications for planetary magnetic sounding via magnetic induction. The magnetotelluric technique (MT) is a passive electromagnetic sounding technique that has been used to great success on Earth and Moon for exploring the subsurface geological structures^15^. It has also been proposed to use MT for subsurface sounding on Mars^16^,^17^ based on the large magnetic field fluctuations in the ionosphere^18^. Our finding of weak and quiet field in atmosphere at Venus will be most probably true for Mars, although Mars’ weaker ionosphere and crustal fields will introduce some differences. In both cases, any magnetotelluric sounding would be best performed from landers rather than from balloons and airplanes. However, a method of using the magnetic fields draped through the ionosphere at low altitudes has recently been suggested^8^. Finally, we note that, at the small field strengths seen by VEX over such a small area and over a single pole, we cannot constrain further the Venus magnetic moment.

## Additional Information

**How to cite this article**: Zhang, T. L. *et al*. Weak, Quiet Magnetic Fields Seen in the Venus Atmosphere. *Sci. Rep*. 6, 23537; doi: 10.1038/srep23537 (2016).

## Figures and Tables

**Figure 1:**
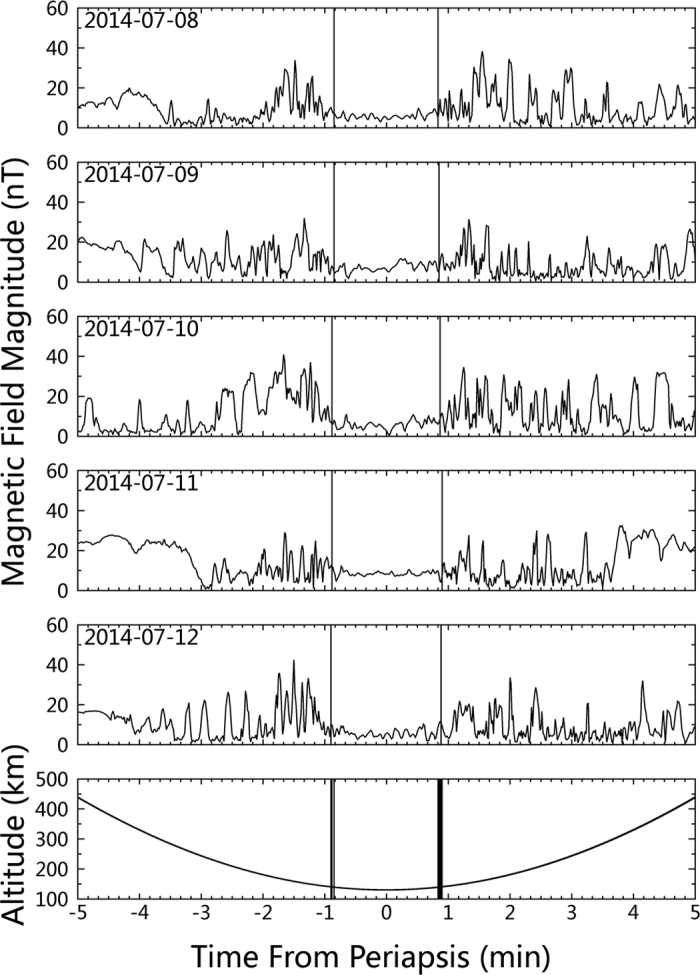
Magnetic field strength as a function of time from periapsis for five consecutive passes during Venus Express aerobraking period. The 10 min data interval is corresponding to an spacecraft trajectory from over 400 km altitude to periapsis around 130 km altitude and back to over 400 km altitude as shown in the lowest panel. Periapsis altitude for these orbits ranged from 129.7 to 130.7 km and solar zenith angle from 93.6° to 95.3°. The 140-km crossing is marked on each panel, and all 5 crossings marked on the lower panel.

**Figure 2:**
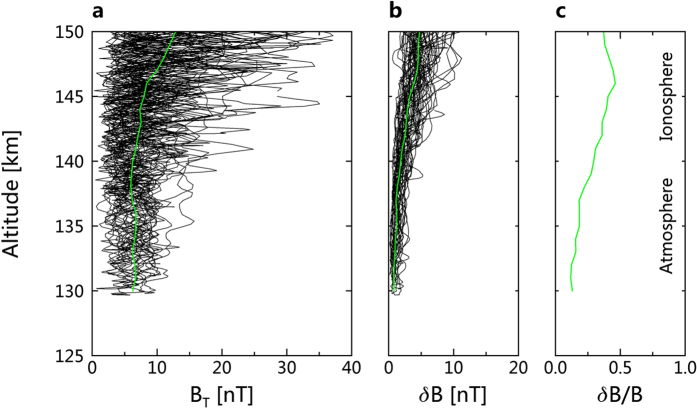
Altitude profiles of magnetic field strength panel (**a**) and fluctuation amplitude panel (**b**) and their ratio panel (**c**). Sixty-six profiles from 33 passes during June 6 to July 12, 2014, when the Venus Express periapsis altitude was below 150 km, are displayed. The field strength data shown in left panel is 1 second resolution and the magnetic field fluctuation δB is indicated as the standard deviation of the field in 12 second bin. The green lines in panels (**a,b**) are medians. The line in panel c is the ratio of these medians.
